# Impact of CNA on AML prognosis

**DOI:** 10.18632/oncotarget.23404

**Published:** 2017-12-18

**Authors:** Fanny Gonzales, Meyling H. Cheok

**Affiliations:** Univ. Lille, Inserm, CHU Lille, UMR-S 1172 - JParc-Centre de Recherche, Jean-Pierre Aubert Neurosciences and Cancer Research Center, Lille, France

**Keywords:** acute myeloid leukemia, copy-number alteration, cytogenetics, prognosis, ERG

Acute myeloid leukemia (AML) is the most frequent acute hematologic malignancy in adults and five-year overall survival rates approach 60% in young adults and children but are only about 10% in the elderly patient population [1, 2]. AML is a heterogeneous disease exemplified by its clinical presentation, biological characteristics and varying degree of clinical response to chemotherapy. Despite this inherent heterogeneity, the therapeutic strategy is based on three prognostic risk groups (favorable, unfavorable or intermediate risk), the latter encompassing two thirds of all patients. This classification, largely based on specific cytogenetic and molecular abnormalities [2], is still suboptimal as prognosis and therapeutic response remain variable within each risk group. The discovery of new prognostic markers is needed to improve treatment stratification and subsequently patient outcomes.

In this respect, we investigated copy number alterations (CNA) as potential new prognostic markers [3]. The underlying hypothesis is that cancer cells are characterized by an accumulation of genetic and genomic alterations [4, 5], CNA could constitute easily detectable prognostic markers, especially in cases of cytogenetic failure, related to technical issues occurring in about 10% of AML. Although some studies have associated presence of CNA with unfavorable prognosis in AML [5, 6, 7], no specific prognostic CNA profile had been identified so far.

CNA were analyzed in paired diagnosis and complete remission bone marrow samples of 119 patients, collected at two French centers, by genome-wide high resolution SNP-array. Secondly, CNA found associated to AML treatment response or prognosis were studied in an independent national cohort of 248 patients (validation cohort) and in 170 samples provided by The Cancer Genome Atlas (TCGA), in order to validate their specificity. Overall, CNA were found in 50% of AML samples and most patients only have one CNA. Deletions were more frequent than amplifications and specific chromosomes were more frequently affected compared to others (i.e., chromosomes 8, 11 and 21 for amplifications and 7, 12, 17 and 21 for deletions).

Four CNA were associated to prognosis: three amplifications (two on chromosome 21 and one on chromosome 11) and one deletion on chromosome 11. The presence of one of these 4 CNA (defined as “CNA marker”) increased mortality by 4 to 5 fold with a mean survival of 1.6 years compared to 5 years for patients with no “CNA marker”. These results were independently confirmed on the validation- and TCGA cohorts.

These four CNA involve a total of 26 genes with various biological functions. In order to identify specific genes implicated in prognosis, mRNA expression at these chromosomal loci was assessed. Notably, the amplification at the 21q22.2 locus was significantly associated to an increase of ETS transcription factor (*ERG*) mRNA expression in the TCGA cohort, for which CNA and gene expression data were available. Moreover, an association between poor overall survival and *ERG* gain was found in all three cohorts.

With regard to AML gene mutations, no association was found between “CNA marker” and gene mutations (i.e., *IDH1/2, DNMT3A, RUNX1, TET2, ASXL1, NPM1, FLT3, CEBPα, MLL-PTD*) or *EVI1* over-expression. However, “CNA marker” was frequently associated with mutant *TP53*, one criteria of unfavorable risk according to ELN classification. Furthermore, AML with mutant *TP53* have a high median number of CNA (8.5 versus 1 with wild-type *TP53*), supporting the association of *TP53* mutation and increased genomic instability, characteristic of complex cytogenetics. Similarly, *TP53* mutation was found in 71% of patients with *ERG* gain.

Multivariate analyses showed that “CNA marker”, *ERG* gain and mutant *TP53* refined current ELN classification. To test the impact of these two criteria on prognosis, we defined two new risk groups:

– a “very unfavorable risk” group, part of the unfavorable risk group including AML cases with “CNA marker” or *TP53* mutation and
– a “unfavorable-like risk” group, part of the intermediate risk group including AML cases with “CNA marker”.

With this refined prognostic classification scheme, 15% and 19% of patients were reclassified. All patients from the “very unfavorable risk group” had a median survival of less than 2 years and outcome tended to be worse in the “unfavorable like” group compared to the intermediate group.

To better understand the mechanisms implied in the prognostic impact of “CNA marker”; we focused on chemotherapy resistance because the incidence of refractory disease was higher in the group with “CNA marker” compared to the group without (41 versus 14%). In particular, an *ERG* gain at the locus 21q22.2 was detected in 80% of refractory patients with “CNA marker”, and our *ex vivo* data confirmed higher resistance to cytarabine in association with *ERG* over-expression; implying that alternative therapies should be used in this group of patients.

In conclusion, this study identified two new prognostic markers: “CNA marker” and *ERG* gain. These specific, robust and universal markers were associated with *TP53* mutation, and could lead to better classification and treatment stratification of AML patients.
